# Dose differences between the three dose calculation algorithms in Leksell GammaPlan

**DOI:** 10.1120/jacmp.v15i5.4844

**Published:** 2014-09-08

**Authors:** Andy (Yuanguang) Xu, Jagdish Bhatnagar, Greg Bednarz, Ajay Niranjan, John Flickinger, L. Dade Lunsford, M. Saiful Huq

**Affiliations:** ^1^ Department of Radiation Oncology University of Pittsburgh Cancer Institute Pittsburgh PA USA; ^2^ Department of Neurological Surgery University of Pittsburgh Medical Center Pittsburgh PA USA

**Keywords:** Gamma Knife, dose calculation, inhomogeneity effect

## Abstract

The purpose of this study was to evaluate the dose differences introduced by the TMR 10 and the convolution dose calculation algorithms in GammaPlan version 10, as compared to the TMR classic algorithm in the previous versions of GammaPlan. Computed axial tomographic images of a polystyrene phantom and a human head were acquired using a GE LightSpeed VCT scanner. A treatment target with a prescription dose of 20 Gy to 50% isodose line was defined in the phantom or the head CT set. The treatment times for single collimator, single shot placements were calculated using the three dose calculation algorithms in GammaPlan version 10. Four comparative studies were conducted: i) the dose matrix position was varied every 10 mm along the x‐, y‐, z‐axes of the stereotactic coordinate system inside the phantom and the treatment times were compared on each matrix for the three collimators of the Gamma Knife Perfexion and the four collimators of the 4C; ii) the study was repeated for the human head CT dataset; iii) the matrix position was varied every 20 mm in the X and the Y directions on the central slice (Z = 100 mm) of the head CT and the shot times were compared on each matrix for the 8 mm collimator of both units; a total of 51 matrix positions were identified for each unit; iv) the above comparison was repeated for the head CT transverse slices with Z = 20, 40, 60, 80, 120, 140, and 160 mm. A total of 271 matrix positions were studied. Based on the comparison of the treatment times needed to deliver 20 Gy at 50% isodose line, the equivalent TMR classic dose of the TMR 10 algorithm is roughly a constant for each collimator of the 4C unit and is 97.5%, 98.5%, 98%, and 100% of the TMR 10 dose for the 18 mm, 14 mm, 8 mm, and the 4 mm collimators, respectively. The numbers for the three collimators of the Perfexion change with the shot positions in the range from 99% to 102% for both the phantom and the head CT. The minimum, maximum, and the mean values of the equivalent TMR classic doses of the convolution algorithm on the 271 voxels of the head CT are 99.5%, 111.5%, 106.5% of the convolution dose for the Perfexion, and 99%, 109%, 104.5% for the 4C unit. We identified a maximum decrease in delivered dose of 11.5% for treatment in the superior frontal/parietal vertex region of the head CT for older calculations lacking inhomogeneity correction to account for the greater percentage of the average beam path occupied by bone. The differences in the inferior temporal lobe and the cerebellum/neck regions are significantly less, owing to the counter‐balancing effects of both bone and the air cavity inhomogeneities. The dose differences between the TMR 10 and the TMR classic are within ± 2.5% for a single shot placement on both Perfexion and 4C. Dose prescriptions based on the experiences with the TMR classic may need to be adjusted to accommodate the up to 11.5% difference between the convolution and the TMR classic.

PACS numbers: 87.55.D, 87.55.kd

## I. INTRODUCTION

The Leksell GammaPlan (LGP) software package^(1)^ (Elekta Instrument AB, Stockholm, Sweden) is a treatment planning platform designed for the stereotactic radiosurgery procedures using the Leksell Gamma Knife units.^2^, ^3^ The LGP planning system performs three‐dimensional radiation dose calculations and dose statistics analyses based on the head geometry of the patient being treated, the treatment shots planned, and the configuration of the Gamma Knife unit. In addition, a set of image processing tools is also included in the LGP to facilitate the target delineation and the interactive treatment planning process.

Several versions of the LGP have been developed in conjunction with the Gamma Knife models U, B, C, 4C, and the Gamma Knife Perfexion. In the earlier versions of the LGP, the patient head is approximated by a semispherical 3D water phantom based on the measurements of 24 predefined points on the patient skull. The LGP system calculates the radiation dose at an arbitrary point in the patient head as the superposition of the dose contributions from all the radiation sources included in the treatment shots placed.^4^, ^5^ The dose contribution from a single radiation source is determined from the water‐based TMR classic algorithm using the strength of the cobalt‐60 source, the coordinates of the cobalt‐60 source and the calculation point, the skull shape information, and a set of predefined beam profiles for the selected collimator. The TMR classic algorithm is generally considered a good approximation for targets located at the center of the brain, but not as accurate when being applied to the peripheral and/or heterogeneous regions.

In recent years, two new dose calculation algorithms, namely the TMR 10^(6)^ and the convolution algorithms,^(7)^ were introduced into the LGP planning system version 10. The TMR 10 algorithm is an evolution of the water‐based TMR classic algorithm with updated physics parameters^(6)^ from more advanced measurements and more accurate Monte Carlo simulations with the PENELOPE code.^8^, ^9^ The updated physics parameters include the beam profile data for the existing Gamma Knife units and all the fitted parameters for single‐source radiation dose calculation. The depth dose formalism for the Gamma Knife B & C was also slightly changed in the TMR 10 algorithm, to be consistent with the formalism for the Perfexion.^(6)^

The development of the CT‐based convolution algorithm^10^, ^11^ was driven by several objectives including a more reliable way of skull shape definition, a more accurate method for scattered dose calculation, and a major improvement for the tissue inhomogeneity dose correction. Initial studies on clinical Gamma Knife treatment plans have indicated that there is a nonnegligible difference between the absolute dose values from the TMR classic algorithm and the Monte Carlo simulations.^12^, ^13^, ^14^ Analyses of the potential effect of the change of the skull shape definition method in Gamma Knife radiosurgery were also undertaken.^(15)^

In this work, we present an evaluation of the dose differences between the TMR classic, the TMR 10, and the convolution algorithm for both the Gamma Knife C models and the Perfexion Gamma Knife units. Advantages and disadvantages of the use of these algorithms for patient dose calculations are also discussed.

## II. MATERIALS AND METHODS

### A. Configuration of the LGP version 10

The LGP version 10 was configured for a Gamma Knife Perfexion and a Gamma Knife 4C at the University of Pittsburgh Medical Center in 2011. The dose rates for the largest collimators of both units were measured using an Exradin A16 ion chamber (Standard Imaging Inc., Middleton, WI) and a 16 cm diameter spherical polystyrene phantom following the TG 21 protocol.^16^, ^17^ To obtain the dose rates in water that are required by the planning system, a mass energy‐absorption coefficient (ॖ/ρ) ratio of 1.036 was applied to the measured dose rates in polystyrene. Following the recommendations from the manufacturer, the output factors used were 1, 0.9, and 0.814 for the 16 mm, 8 mm, and 4 mm collimators of the Perfexion unit, and 1, 0.985, 0.956, and 0.881 for the 18 mm, 14 mm, 8 mm, and 4 mm collimators of the 4C, respectively.

**Figure 1:**
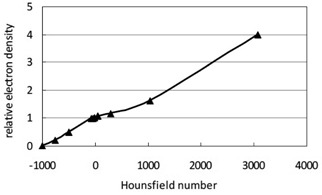
CT density calibration curve for the GE LightSpeed VCT scanner measured using the CIRS model 062A electron density phantom.

In order to obtain the CT calibration curve for the convolution algorithm, a CIRS model 062A electron density phantom (CIRS Inc., Norfolk, VA) with 11 inserts of predefined electron density values was scanned using a GE LightSpeed VCT scanner (GE Medical Systems Inc., Waukesha, WI). Two hundred axial images with a slice thickness of 1.25 mm were acquired for this phantom using a 120 kVp head CT protocol. Five Hounsfield numbers were taken from the central region of each insert and an averaged Hounsfield number was calculated. A one‐to‐one correspondence between the averaged Hounsfield numbers and the predefined relative electron density values was then established for the scanner. Figure 1 shows the plot of the CT calibration curve that was created in the planning system for the GE LightSpeed VCT scanner. Table 1 gives the relative electron densities (relative to water) and the corresponding equivalent materials for the 11 inserts used to obtain the CT calibration curve.

**Table 1:** Relative electron densities and equivalent materials of the 11 inserts used for the CT electron density calibration

*Relative Electron Density*	*Equivalent Material*
0	Air
0.2	Lung (inhale)
0.5	Lung (exhale)
0.97	Adipose
0.99	Breast
1	Water
1.06	Muscle
1.07	Liver
1.16	Trabecular bone
1.61	Dense bone
3.98	Titanium rod

### B. Dose calculations in a Polystyrene phantom

**Figure 2:**
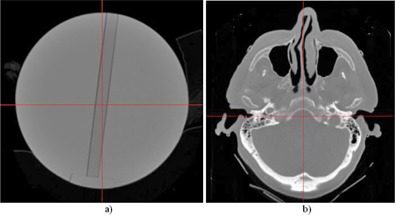
Sample transverse images of the electron density maps for (a) the polystyrene phantom and (b) the head CT.

To study the differences between the three dose calculation algorithms in a uniform medium, the spherical polystyrene phantom used for the dose rate measurements was scanned using the same head CT protocol as for the electron density phantom. Prior to scanning, a solid polystyrene insert was placed in the dosimetry phantom to minimize dosimetric uncertainties caused by air cavities and air gaps from the ion chamber or film inserts. The 120 transverse CT slices acquired were imported into the LGP 10 and the stereotactic coordinate system was defined such that the phantom center had the coordinates of 100 mm, 100 mm, and 100 mm. The skull shape for the phantom was generated using the imaging skull definition method and manually edited with the paint brush tool. A 3D electron density map was then created for the phantom using the CT density calibration curve defined in the planning system. A sample transverse slice of the electron density map for the polystyrene phantom is shown in Fig. 2(a).

After a treatment plan was created for the Perfexion unit, a dose calculation matrix was defined on the CT image set with a matrix center position of 100 mm, 100 mm, and 100 mm, a grid size of 0.4 mm, and a dose prescription of 20 Gy to the 50% isodose line. Dose calculations were performed using both the TMR 10 and the convolution algorithms for a single 4 mm shot with a gamma angle of 90° and a shot position of 100 mm, 100 mm, and 100 mm. The resultant treatment times from both algorithms were then recorded along with the coordinates of the reference points and the equivalent doses from the TMR classic algorithm. The equivalent TMR classic dose for each matrix is the dose at the reference point from the TMR classic algorithm for the same machine configuration, the same patient geometry, and the same shot arrangement from a TMR 10 or convolution calculation for a certain prescription dose. This information is provided in LGP version 10.1 for all the calculation matrices at the time of a TMR 10 or a convolution calculation.

To evaluate the differences between the three algorithms in different regions of the phantom, the dose matrix position and the shot position were varied concurrently every 10 millimeter along the x‐, y‐, z‐axes of the stereotactic coordinate system. A total of 15 matrix positions and shot positions were studied on each axis by varying the coordinate values from 20 to 160.

For a comparison of the three dose calculation algorithms with different collimators, the above procedure was repeated for the other six collimators of the Perfexion and the 4C units with grid sizes of 0.4 mm, 0.8 mm, 1.4 mm, 1.6 mm, and 1.8 mm for the 4 mm, 8 mm, 14 mm, 16 mm, and 18 mm collimators, respectively.

### C. Dose calculations in a human head CT

A human head CT image set with 120 axial slices and 1.25 mm slice thickness was selected to perform a comparative study of the three algorithms for real patient geometry. The CT series was acquired using the same GE CT scanner following the same protocol as used for the phantom study. The procedures for the skull definition and the electron density calibration were also the same. A sample transverse slice of the electron density map for the head CT series is shown in Fig. 2(b).

In addition to the one‐dimensional studies performed for the phantom, 2D and 3D comparisons of the dose differences between the three algorithms were also made for the head CT. For the 2D comparison, the transverse slice at Z = 100 mm and the 8 mm collimator from both machines were used. The dose calculation matrix positions and the shot positions were varied every 20 mm along the X and the Y directions on the transverse slice. The data points from the failed convolution calculations inside air or bone inhomogeneity were excluded from the study. A total of 51 matrix positions were identified for each machine.

For the 3D study, the 8 mm collimators from both Gamma Knife units were used. The 2D comparison was repeated every 20 mm for the transverse slices ranging from Z = 20 mm to Z = 160 mm. The number of the valid shot positions on the eight transverse slices was 5, 30, 45, 40, 51, 33, 31, and 36, respectively. A total of 271 matrix positions were studied.

## III. RESULTS

### A. Treatment time

**Figure 3:**
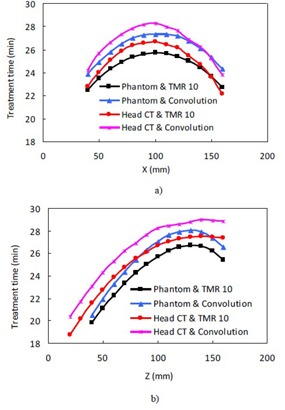
Treatment times from the TMR 10 and the convolution algorithms for the Perfexion 8 mm shots with a prescription dose of 20 Gy to 50% isodose line: a) shots on the x‐axis; b) shots on the z‐axis.

Figure 3(a) shows the treatment times from the TMR 10 and the convolution algorithms for the Perfexion 8 mm shots placed on the x‐axis of the phantom and the head CT with a prescription dose of 20 Gy to the 50% isodose line. The treatment time from the convolution algorithm is consistently longer than that from the TMR 10 algorithm for the same matrix position. The treatment time from the same algorithm is in general larger in the central region than in the peripheral regions. This is consistent with the behavior of the sum of two exponential attenuation functions with a fixed total attenuation depth. In Gamma Knife radiosurgery, the multiple radiation sources can be roughly viewed as two groups of radiation sources opposite to each other. The maximum attenuation of the radiation beams occurs when a spherical phantom is centralized and the attenuation depths from all sources are equal.

Shown in Fig. 3(b) are the treatment times along the z‐axis of the stereotactic coordinate system for the phantom and the head CT on the Perfexion. The treatment time increases with increasing Z coordinate, which is consistent with the fact that more beams are arranged to come from the vertex side of the collimator system in the Leksell Gamma knife units.

The curves representing the treatment times for the other collimators of the Perfexion and the 4C units are similar in shape and are not presented here. It should be pointed out that the reference points used for dose calculation in a matrix are not necessarily the same for the TMR 10 and the convolution algorithms. By definition, the LGP system chooses the point of maximum dose from each calculation matrix as the dose reference point. The maximum dose points are not necessarily the same even for a single collimator shot, considering the completely different formalism used in the two algorithms. However, our observation was that the reference point positions from the two calculations usually agree to within one or two pixels for the same matrix. The change of the dose values within one or two pixels should be small in the central regions of the radiation fields.

### B. Dose difference between TMR 10 and TMR classic

**Figure 4:**
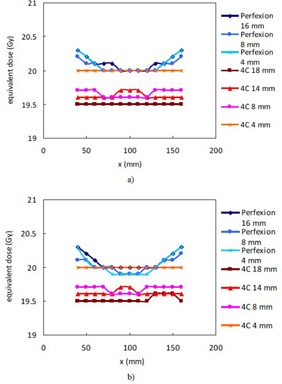
Equivalent TMR classic doses for the TMR 10 algorithm for single collimator shots placed on the x‐axis with a prescription dose of 20 Gy: a) shots placed in the phantom; b) shots placed in the head CT.

Figure 4 compares the equivalent TMR classic doses of 20 Gy in TMR 10 for single collimator shots placed at varying positions on the x‐axis of the polystyrene phantom and the head CT. The shape of the curves for the same collimator from Fig. 4(a) (the phantom data) and Fig. 4(b) (the head CT data) are similar. The equivalent TMR classic dose for 20 Gy in TMR 10 is roughly a constant for each collimator of the 4C unit and is 19.5 Gy (97.5%), 19.7 Gy (98.5%), 19.6 Gy (98%), and 20 Gy (100%) for the 18 mm, 14 mm, 8 mm, and 4 mm collimator, respectively. The corresponding values for the three collimators of the Perfexion unit fluctuate in the range from 19.9 Gy (99.5%) to 20.4 Gy (102%), as a result of the asymmetric arrangement of the radiation sources in the Perfexion and the ring‐specific beam profiles used in the TMR 10 algorithm.

**Figure 5:**
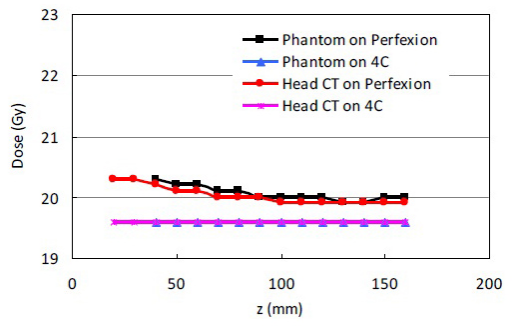
Equivalent TMR classic doses from the TMR 10 algorithm for single 8 mm shots placed on the z‐axis with a prescription dose of 20 Gy.

Figure 5 plots the equivalent TMR classic doses for single 8 mm shots placed on the z‐axis from the TMR 10 algorithm for a prescription dose of 20 Gy to 50%. There is a slight increase in the equivalent TMR classic dose with decreasing Z coordinate on the Perfexion unit for both the phantom and the head CT. The curves for the other collimators are similar in shape and are not shown here.

### C. Dose difference between convolution and TMR classic

**Figure 6:**
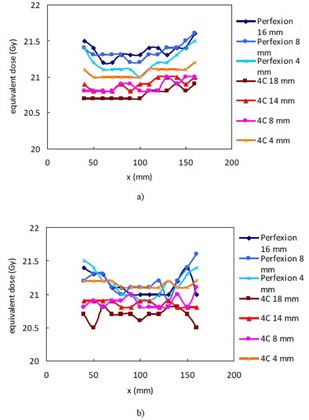
Equivalent TMR classic doses from the convolution algorithm for single collimator shots placed on the x‐axis with a prescription dose of 20 Gy: a) shots in the phantom; b) shots in the head CT.

Figure 6 compares the equivalent TMR classic doses of 20 Gy in convolution for single collimator shots placed at varying positions on the x‐axis of the polystyrene phantom and the head CT. As could be expected, the variations in the equivalent TMR classic doses with the X coordinate are larger for both units when compared to the corresponding variations for the TMR 10 in Fig. 4. For the head CT, the larger variations are simply the result of taking into account the tissue inhomogeneity in the convolution calculation. For the phantom, the TMR 10 treats the polystyrene phantom as a perfect water sphere, whereas the convolution algorithm picks up the electron density fluctuations caused by the residue air cavities and air gaps inside the phantom. The equivalent TMR classic doses in Fig. 6(b) fluctuate more than the doses in Fig. 6(a) because of a more pronounced electron density inhomogeneity in the head CT.

**Figure 7:**
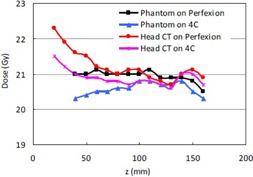
Equivalent TMR classic doses from the convolution algorithm for single 8 mm shots placed on the z‐axis with a prescription dose of 20 Gy.

Figure 7 shows the equivalent TMR classic doses for single 8 mm shots placed on the z‐axis from the convolution algorithm. An interesting phenomenon can be observed in the curves for the head CT on both units. As the Z coordinate decreases from the central point at 100 mm, the equivalent TMR classic dose starts to increase gradually and reach a maximum at Z = 20 mm, where the treatment shots are in the superior frontal/parietal vertex region. This behavior is not seen in the phantom data in Fig. 7 and the corresponding curves for the TMR 10 in Fig. 5.

The large difference between the convolution and the TMR classic algorithms for treatment shots in the superior frontal/parietal vertex region can be attributed to the high radiation attenuation in the dense skull bone. As the treatment shots approach the frontal/parietal bone, the total attenuation depths of the radiation beams (especially for the beams from the anterior side of the Gamma Knife units) in the patient head decreases. The percentage of the attenuation depth in the frontal bone increases. The contribution from the high radiation attenuation in the dense bone starts to play an important role in the overall dose calculation and could cause an 11.5% dose difference for Perfexion (7.5% for 4C) between the convolution and the TMR classic.

**Figure 8:**
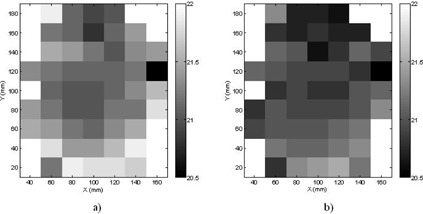
Equivalent TMR classic doses from the convolution algorithm for single 8 mm shots placed on the Z = 100 cm slice of the head CT with a prescription dose of 20 Gy: a) shots from the Perfexion; b) shots from the 4C.

Figure 8 shows the equivalent TMR classic doses for the 8 mm shots placed on the Z = 100 mm slice of the head CT with a prescription dose of 20 Gy. The two‐dimensional dose maps (20 mm × 20 mm pixel size) for the Perfexion (Fig. 8(a)) and the 4C (Fig. 8(b)) follow a similar pattern. The white spots around the corners of the pictures are regions outside the patient skull. The equivalent TMR classic dose is in general smaller in the central region of the slice than in the peripheral region, even though fluctuations caused by the local bone or air inhomogeneities can be observed. For the same matrix position, the equivalent TMR classic dose from the 4C is in general smaller than that from the Perfexion.

**Figure 9:**
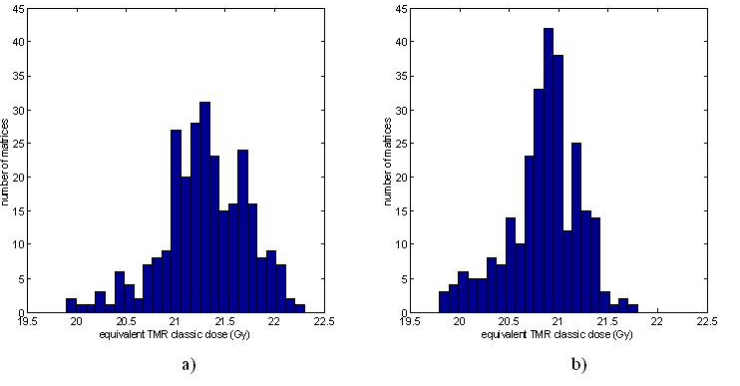
Histogram of the equivalent TMR classic doses for the convolution algorithm for single 8 mm shot placed on the 20 mm × 20 mm × 20 mm voxels of the head CT with a prescription dose of 20 Gy: a) shots from the Perfexion; b) shots from the 4C.

Figure 9 plots the histogram of the equivalent TMR classic doses for the convolution algorithm for the 8 mm shots placed on the 2 cm × 2 cm × 2 cm voxels of the head CT with a prescription dose of 20 Gy. A total of 271 shots were included in the histogram analysis for each unit. The minimum, maximum, and the mean of the equivalent TMR classic doses are 19.9 Gy (99.5%), 22.3 Gy (111.5%), and 21.3 Gy (106.5%) for the Perfexion. The corresponding values for the 4C units are 19.8 Gy (99%), 21.8 Gy (109%), and 20.9 Gy (104.5%), respectively. The voxels of high doses are usually seen in the superior frontal/parietal vertex region or close to bone inhomogeneity. The low‐dose regions are primarily in the inferior temporal lobe and the cerebellum/neck regions or around air cavities.

## IV. DISCUSSION

The dose prescription/calculation process is an important part of a Gamma Knife radiosurgery procedure. As is the case with other modalities of radiation therapy, the dose calculation algorithms and associated software packages in Gamma Knife radiosurgery have been updated periodically.

The development of the water‐based TMR classic algorithm for Gamma Knife radiosurgery dose calculation stemmed from the unique properties of the Leksell Gamma Knife machines, including the small dimension of the radiation fields and the mono‐energetic nature of the cobalt‐60 sources. The water‐based TMR classic algorithm has been widely used, to date, with proven clinical outcomes, which provides a solid basis for future improvements.

The TMR 10 algorithm was introduced as an improvement over the TMR classic with updated physics parameters and dose calculation models. In this study, no clinically significant differences were observed between the TMR 10 and the TMR classic calculations for a polystyrene phantom and a human head CT for both the Perfexion and the 4C Gamma Knife units. This is consistent with the observations from other investigators.^(6)^

The results from the studies on the convolution algorithm can be interpreted in three different ways. First, a maximum dose difference of 11.5% has been found in this study between the convolution and the TMR classic algorithms for the Perfexion treatment shots placed in the superior frontal/parietal vertex regions of a human head CT (9% for the 4C units). The prescription doses for the treatments of the diseases in these regions with the TMR algorithms may need to be adjusted higher to account for the high attenuations from the vertex bones.

Second, the dose difference between the convolution and the TMR classic algorithms varies from ‐0.5% to 11.5% for the Perfexion (‐1% to 9% for the 4C), depending on the location of the treatment shots placed in the head CT used in this study. This indicates that a considerable tissue inhomogeneity effect might be associated with the Gamma Knife treatments around dense bones or air cavities.^(13)^ Therefore, implementation of the convolution algorithm might be necessary for optimal results in routine Gamma Knife treatments.

Third, an average dose difference of 6.5% has been found in this study for the Perfexion (4.5% for the 4C) between the convolution and the TMR classic algorithm for the 271 treatment shots placed on the head CT. Implementation of the convolution algorithm for Gamma Knife radiosurgery treatment will thus need to be followed with careful monitoring of outcome studies. Even though the convolution algorithm might be more advanced and accurate, historically the dose prescription guidelines for Gamma Knife radiosurgery have evolved based on the outcome studies associated with the TMR classic dose calculation algorithm.

The results obtained for the Perfexion and the 4C units in this study are similar in nature, even though quantitatively there are some small differences. It should be noted that the present work does not employ any treatment plans with multiple shots or composite shots. Furthermore, only a single set of head CT is used in the model study. For an in‐depth understanding of the differences between the three dose calculation algorithms, systematic studies on the clinical treatment plans for a variety of disease sites will be needed in the future.

## V. CONCLUSIONS

In this paper we report the results from a preliminary study of the dose differences between the three dose calculation algorithms in Leksell GammaPlan version 10 performed using a polystyrene phantom and a human head CT. The difference between the TMR 10 and TMR classic are demonstrated to be within ± 2.5% for all the collimators of a Perfexion and a 4C unit. For calculations performed on the 271 voxels of a head CT, the convolution algorithm yields a minimum, maximum, and mean dose values that is 99.5%, 111.5%, and 106.5% of the TMR classic dose. The corresponding values for the 4C unit are 99%, 109%, and 104.5%, respectively.

A maximum decrease of 11.5% in delivered dose was observed for the treatment in the superior frontal/parietal vertex region using the TMR classic calculation. The TMR classic calculation does not incorporate tissue inhomogeneity correction to account for the greater percentage of the average beam path travelled in bone. The differences in the inferior temporal lobe and the cerebellum/neck regions are significantly less, owing to the counter‐balancing effects of both bone and the air cavity inhomogeneities. Dose prescriptions based on the experiences with the TMR classic algorithm may need to be adjusted to accommodate the up to 11.5% difference between the convolution and the TMR classic algorithms.

## Supporting information

Supplementary Material
