# Supplementary material for: Dose differences between the three dose calculation algorithms in Leksell GammaPlan
Source: J Appl Clin Med Phys. 2014 Sep 8;15(5):89–99. doi: 10.1120/jacmp.v15i5.4844 (PMC5711097; doi:10.1120/jacmp.v15i5.4844)
Supplement: Supplementary file 1 — Supplementary Material [file ACM2-15-089-s001.doc]

**Dose differences between the three dose calculation algorithms in Leksell GammaPlan®**

**Y. Xu, J. Bhatnagar, G. Bednarz, A. Niranjan, J. Flickinger, L. D. Lunsford, and M. Saiful Huq**

*Department of Radiation Oncology, University of Pittsburgh Cancer Institute, Pittsburgh, PA 15232*

*Andyxu55@gmail.com*

*Department of Neurological Surgery, University of Pittsburgh Medical Center, Pittsburgh, PA 15213*

Running title: Gamma Knife dose calculation
